# Puerarin attenuates cisplatin-induced rat nephrotoxicity: The involvement of TLR4/NF-κB signaling pathway

**DOI:** 10.1371/journal.pone.0171612

**Published:** 2017-02-09

**Authors:** Xu Ma, Lei Yan, Qing Zhu, Fengmin Shao

**Affiliations:** Department of Nephrology, People’s Hospital of Zhengzhou University, Zhengzhou, People’s Republic of China; National Institutes of Health, UNITED STATES

## Abstract

Puerarin was a major isoflavonoid derived from the Chinese medical herb *radix puerariae* (Gegen). In present study effect of puerarin on cisplatin nephrotoxicity was evaluated. Rat model of nephrotoxicity was established by a single intraperitoneal injection of cisplatin (7mg/kg). Puerarin was administrated through caudal vein injection once per day at the dose of 10mg/kg, 30mg/kg and 50mg/kg. Biochemical assays showed that after cisplatin treatment the serum urea and creatinine increased significantly compared with control (P<0.05). Cisplatin treatment significantly increased xanthine oxidase (XO) activity and malondialdehyde (MDA) formation, and significantly decreased the levels and /or activities of enzymatic and non-enzymatic antioxidants (GSH, GPx, GST, GR, SOD, CAT), in the kidney tissues. Renal levels of TNF-α and IL-6, two important inflammatory cytokines, were also upregulated by cisplatin. Histopathological examination indicated that cisplatin treatment resulted in severe necrosis and degeneration, hyaline casts in the tubules, intertubular hemorrhage, congestion and swelling in glomerulus and leukocytes infiltration in the kidney tissues. Western blot results demonstrated that cisplatin increased TLR4 and NF-κB protein expression in the kidney tissues. However, all these changes induced by cisplatin were significantly attenuated by puerarin treatment in dose-dependent manner, which indicated the renal protective effect of puerarin. Cell culture experiments illustrated that puerarin alone treatment concentration-dependently inhibited COLO205 and HeLa tumor cell growth and dose-dependently promoted the antitumor activity of cisplatin in COLO205 and HeLa tumor cells. The promotion effects might be attributed to suppression of cisplatin-increased NF-κB p65 expression by puerarin. Taken together, findings in this study suggested that puerarin exhibited renal protection against cisplatin nephrotoxicity via inhibiting TLR4/NF-κB signaling, with no inhibition but promotion effect on the antitumor activity of cisplatin. Puerarin might be a promising adjuvant agent for cisplatin chemotherapy.

## Introduction

As one of most potent anti-tumor drugs, cisplatin was used for treatment of a wide variety of solid tumors including testicular, ovarian, bladder, endometrial, cervical, and lung cancers [1–5]. However, due to the severity of side toxicities including nephrotoxicity, clinical use of cisplatin was constrained in 25%–35% of the hospitalized patients undergoing chemotherapy [6]. Owing to the fact that cisplatin exerted potent antitumor activity and couldn’t be abandoned at present in clinic, an urgent demand existed for cancer researchers to develop new adjuvant therapy to ameliorate toxicities of cisplatin without inhibitory effects on the antitumor activity of cisplatin. Recently, phytochemicals attracted more and more eyeballs of cancer scientists [7, 8] and some herbal compounds have been studied to attenuate cisplatin nephrotoxicity [6, 9–12], which might potentially help to expand the clinical application of cisplatin.

Puerarin, a major isoflavonoid derived from the Chinese medical herb *radix puerariae* (Gegen), has been reported to harbor a wide spectrum of pharmacological properties including antioxidant and anti-inflammatory, present many beneficial effects on various medicinal purposes including treating neuronal disease, cardiovascular and cerebrovascular disease [13, 14]. Recent investigations showed that puerarin had *in vitro* and *in vivo* antitumor activities through induction of mitochondria-mediated apoptosis pathway [13, 15–17], which demonstrated that puerarin might be a potential antitumor candidate chemical and was worthy of more investigations. What’s more, Wang L. *et al* reported that puerarin provided renal protection against lead-induced nephrotoxicity in rat through regulating apoptosis and autophagy in proximal tubular cells [18–21]. Nevertheless, so far, no reports existed about the pharmacological effect of puerarin on cisplatin nephrotoxicity. The present study was aimed to answer this question, which would help to justify the future clinical application of puerarin in cancer treatment in combination with cisplatin as adjuvant therapy to reduce toxicities. Furthermore, the molecular mechanisms of puerarin action were also investigated in this study.

## Materials and methods

### Animals

Male Sprague-Dawley rats weighing about 200 grams were purchased from Vital-River (Beijing, China). Animals were maintained at a constant temperature (22 ± 2℃), humidity (55%), and light-dark conditions (12/12 h light/dark cycle). Animals were acclimatized from three days before experiments and were fed with food and tap water *ad libitum*. Animals were cared according to the Guide for the Care and Use of Laboratory Animals published by the National Research Council of the National Academies. The protocol of present study was checked and approved by the animal ethics committee at the People's Hospital of Zhengzhou University.

### Experimental design

Twenty-eight rats were used in this study (n = 7/group). According to previous report [22], cisplatin-induced nephrotoxicity was performed by a single intraperitoneal (i.p.) cisplatin injection (7mg/kg in 0.9% saline). Puerarin was purchased from Sigma-Aldrich (St Louis, MO, USA), dissolved in 0.9% saline and administrated through caudal vein injection once per day. The dose of puerarin used in this study was chosen based on previous report [14].The rats were grouped as follows:

1. Control group: Rats were intraperitoneally injected with 0.9% saline without cisplatin (once per day).
2. Cisplatin group: Cisplatin (Sigma-Aldrich Co, St Louis, MO, USA) in 0.9% saline was injected on the third day of study in a 7mg/kg dose, intraperitoneally.
3. Cisplatin+puerarin group: Three days before cisplatin treatment 10mg/kg, 30mg/kg or 50mg/kg puerarin in 0.9% saline was administrated through the caudal vein. And cisplatin was administrated as in the cisplatin group. Thereafter, puerarin administration was continued for another five days.

At day 5 after cisplatin administration the rats were decapitated under anesthesia. Kidneys were removed and stored at -80℃ for further analysis. Blood samples were collected for serum urea and creatinine measurements.

### Biochemical assays of oxidative stress

Malondialdehyde (MDA) levels in the kidney tissues were determined using the method reported by Utley et al., in 1967 [23]. Reduced glutathione (GSH) content, glutathione reductase (GR), glutathione peroxidase (GPx), glutathione-S-transferase (GST), superoxide dismutase (SOD), catalase (CAT) and xanthine oxidase (XO) activities in the kidney tissues were measured according to the methods as previously reported [1, 24, 25]. Serum creatinine and urea levels were determined using commercially available kits (Nanjing Jiancheng, Nanjing, China).

### Histopathology of kidney tissues

Kidney tissues were fixed in 10% formalin solution and embedded in paraffin. 4 um thick sections were cut and stained with hematoxylin-eosin (H.E) according to standard method. The sections were examined under light microscope. The necrosis and degeneration were determined in 10 randomly selected areas under 20× magnification and graded as follows according to the percentage of necrosis and degeneration: none (score: 0) = normal, mild (score: 1) = <10%, moderate (score: 2) = 10% ~ 25%, severe (score: 3) = 26% ~ 75% and extremely severe (score: 4) = >75%, as previously reported [26].

### Determination of TNF-α and IL-6 levels in kidney tissues

Kidney tissue homogenate of 100 mg protein per milliliter was prepared with phosphate buffer saline (50 mM, pH 7.4) containing 1% protease inhibitor cocktail (Sigma-Aldrich Co, St Louis, MO, USA). After centrifugation at 4000 ×g for 20 min at 4℃, the supernatant was collected and used for measurement of TNF-α and IL-6 with commercially available kits from R&D Systems, USA. The principle of the assays was sandwich ELISA. Absorbance was taken at 450 nm. TNF-α and IL-6 levels in the supernatant were expressed as pg / mg protein.

### Western blot analysis

Protein was extracted from rat kidney tissues and collected cultured cells using Radio Immunoprecipitation Assay (RIPA) Lysis Buffer (Beyotime Institute of Biotechnology, Nanjing, China). Total protein concentration was measured by BCA procedure (Cat: 23225, Pierce, Rockford, IL, USA). About 100ug protein was separated by 10% sodium dodecyl sulfate polyacrylamide gel electrophoresis (SDS-PAGE) and transferred onto a 0.4 μm- polyvinylidene fluoride (PVDF) membrane (Millipore, Bedford, MA, USA). Then the membranes were blocked in 5% defatted milk in Tris-buffered saline containing 0.1% Tween-20 (TBST) for 1 hour at room temperature, washed twice with TBST and incubated with mouse anti-TLR4 monoclonal antibody (1: 500 dilution, Cat: ab89455, Abcam, Shanghai, China), rabbit anti-NF-κB p65 monoclonal antibody (1:1000 dilution, Cat: 8242, Cell Signaling Technology, Denver, Colorado, USA) and mouse anti-β-actin monoclonal antibody (1:1000 dilution, Cat: MAB1501, Millipore, Bedford, MA, USA) at 4°C overnight. After washing with TBST, membranes were incubated with goat anti-rabbit or goat anti-mouse secondary antibody (1:20000 dilution, Jackson ImmunoResearch, Baltimore, Maryland, USA) at room temperature for 1 hours. Membranes were developed with SuperSignal Western reagent (Cat: 34080, Pierce, Pierce, Rockford, IL, USA). Protein bands were imaged using a GelDoc XR System (Bio-Rad, Shanghai, China). The density of protein bands was quantified and analyzed using the free software of Image J (http://rsb.info.nih.gov/ij/).

### Evaluation of tumor cell proliferation

In order to determine whether puerarin interfere in the antitumor activity of cisplatin, in vitro human colon and cervical tumor cell experiments were performed. The dose of puerarin (1–5μg/ml) was chosen based on previously published methods [15]. Human colon cancer cell line COLO205 and cervical cancer cell line HeLa were bought from American Type Culture Collection (ATCC, Manassas, VA, USA). All the cells were cultured in DMEM (Gibco-BRL, Gasthersburg, MD, USA) supplemented with 10% fetal bovine serum (Gibco-BRL, Gasthersburg, MD, USA), 2% penicillin/streptomycin (10,000 U/ml penicillin, 10 mg/ml streptomycin) and grew under the humidified condition of 37°C, 5% CO2 and 95% air atmosphere. When evaluating tumor cell proliferation, 2×10^4^ cells in 200 μl medium per well were seeded in a 96-well plate. Next day, cells were treated by cisplatin in the presence or absence of puerarin for another 24 hours. Then, 20 μl CCK-8 reagent (Dojindo, Kumamoto, Japan) were added to each well and the plate was incubated at 37℃ for additional 2 hours. At last the optical density at 450nm (OD_450nm_) was determined. The cell viability was expressed at percent of the control group.

### Statistical analysis

The statistical analysis was done by using SPSS 18.0 software (SPSS Inc., Chicago, IL, USA). One-way analysis of variance (one-way ANOVA) followed by post hoc Tukey’s test was performed to detect the difference among different groups. P<0.05 was considered as statistically significant.

## Results

### Effect of puerarin on cisplatin-induced renal injury

Table 1 showed that, compared with control cisplatin treatment induced significant increases in serum urea (from 6.81±0.73 mmol/L to 59.24±16.98 mmol/L, P<0.05) and creatinine (from 34.18±6.83 μmol/L to 412.52±128.97 μmol/L, P<0.05). All these changes induced by cisplatin were inhibited by puerarin in a dose-dependent manner. At the dose of 50mg/kg puerarin nearly reversed serum urea and creatinine to normal levels. Compared with control puerarin alone treatment showed no substantial influence on serum biomarkers of renal function.

**Table 1:** Effects of puerarin on serum urea and creatinine levels in rats with cisplatin-induced nephrotoxicity.

Parameters\Groups	Con.	Puer. (50mg/kg)	Cispt	Cispt+10mg/kg Puer.	Cispt+30mg/kg Puer.	Cispt+50mg/kg Puer.
Urea(mmol/L)	6.81±0.73	7.12±0.41^a^	59.24±16.98^b^	52.49±10.61^c^	38.36±9.12^d^	15.43±11.37^d^
Creatinine(umol/L)	34.18±6.83	38.81±5.79^a^	412.52±128.97^b^	397.48±81.69^c^	261.19±79.33^d^	81.89±58.91^d^

^a^ P>0.05 *vs*. Control group

^b^ P<0.05 *vs*. Control group

^c^ P>0.05 *vs*. Cisplatin group

^d^ P<0.05 *vs*. Cisplatin group.

***Note*:** Con.: control; Cispt: cisplatin; Puer.: Puerarin.

### Effect of puerarin on cisplatin-induced oxidative stress in kidney tissues

**Fig 1:**
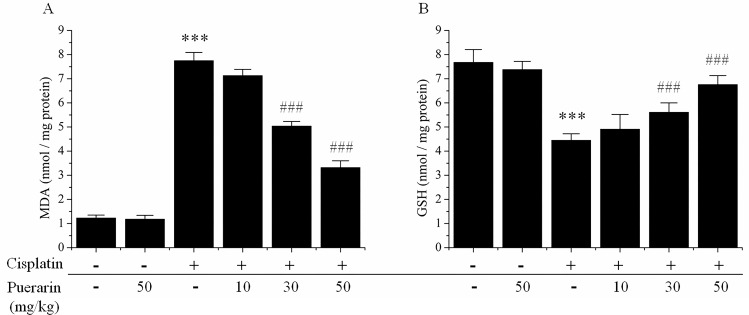
**Effects of puerarin on changes of (A) malondialdehyde (MDA) levels and (B) glutathione (GSH) content in the kidney tissues of cisplatin-treated rats.** ***P<0.05 versus control group; ^###^P<0.05 versus cisplatin alone treated group.

As indicated in Fig 1A, in contrast to control cisplatin administration induced a statistically significant increase (P<0.05) in malondialdehyde (MDA) formation, the biomarker of lipid peroxidation. Compared with control cisplatin treatment rendered a statistically significant decrease (P<0.05) in non-enzymatic antioxidant, reduced glutathione (GSH) content in kidney tissues (Fig 1B). All these changes in kidney tissues induce by cisplatin were dose-dependently reversed by puerarin treatment (P<0.05) (Fig 1). Puerarin alone treatment presented no substantial influences on MDA formation and GSH content in kidney tissues (Fig 1).

**Fig 2:**
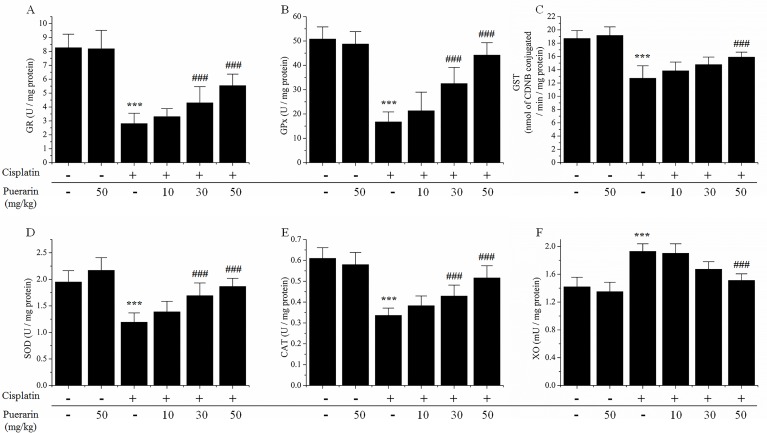
Effects of puerarin on changes of oxidative biomarkers in kidney tissues of cisplatin-treated rats. (A) glutathione reductase (GR) activities; (B) glutathione peroxidase (GPx) activities; (C) glutathione-S-transferase (GST) activities; (D) superoxide dismutase (SOD)activities; (E) catalase (CAT) activities; (F) xanthine oxidase (XO) activities. ***P<0.05 versus control group; ^###^P<0.05 versus cisplatin alone treated group.

Fig 2 showed that the enzymatic anti-oxidants (GR, GPx, GST, SOD, CAT) activities in kidney tissues were significantly suppressed by cisplatin treatment in contrast to control (P<0.05). While the enzymatic oxidant activity, including xanthine oxidase (XO) in kidney tissues was significantly enhanced by cisplatin administration. However, all these changes induced by cisplatin were significantly inhibited by puerarin treatment in a dose-dependent manner (P<0.05), and for some parameters, the changes induced by cisplatin were almost completely reversed by 50 mg/kg puerarin treatment.

### Effect of puerarin on cisplatin-induced renal histopathological alternations

**Fig 3:**
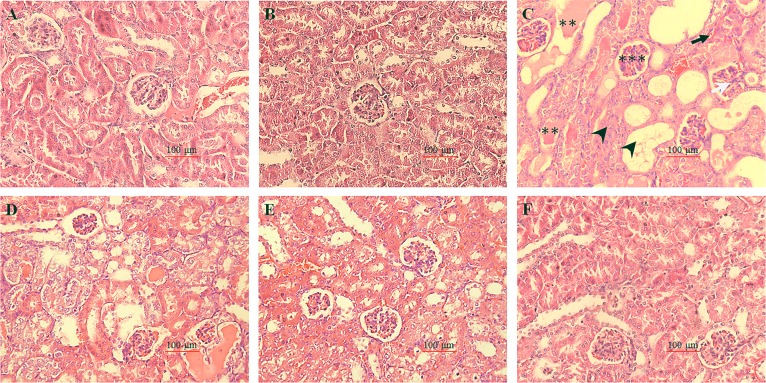
Photomicrographs of rat kidney sections stained with hematoxylin and eosin (H.E) (200×). (A) Kidney sections from control group showed normal morphological view; (B) Kidney tissues section from puerarin alone treated rats (50mg/kg) showed normal morphological view. (C) Kidney tissue section from cisplatin alone treated rats (7 mg/kg) showed severe tubular degeneration and necrosis, hyaline casts in the tubules, intertubular hemorrhage, congestion and swelling in glomerulus; (D-F) Sections from puerarin plus puerarin treated rats. The respective dose of puerarin was (D) 10mg/kg, (E) 30mg/kg and (F) 50 mg/kg. Kidney sections (F) from cisplatin plus 50mg/kg puerarin treated rats showed predominantly normal renal histology with occasional degenerative changes when compared with cisplatin alone treated rats. Black arrow head indicates necrosis and degeneration; Black arrow indicates intertubular hemorrhage; White arrow indicates leukocytes infiltration; Two asterisks indicate hyaline casts in the tubules. Three asterisks indicate congestion and swelling in glomerulus.

Histopathological alternations were illustrated in Fig 3. Kidney sections in control group and puerarin alone treatment group showed normal morphology (Fig 3A and 3B). While in cisplatin group severe necrosis and degeneration, hyaline casts in the tubules, intertubular hemorrhage, congestion and swelling in glomerulus and infiltration of leukocytes were observed (Fig 3B). However, all these phenomenons caused by cisplatin treatment were dose-dependently suppressed by puerarin treatment in cisplatin + puerarin group (Fig 3 and Table 2). What’s more, when the dose of puerarin was 50mg/kg the histological alternations of kidney tissues resulted from cisplatin treatment were almost completely ameliorated (Fig 3F).

**Table 2:** Assessment of the necrosis and degeneration.

Alternations/Groups	Con.	Puer. (50mg/kg)	Cispt	Cispt+10mg/kg Puer	Cispt+30mg/kg Puer	Cispt+50mg/kg Puer.
Necrosis and degeneration	0.00±0.00	0.00±0.00	3.48±0.26^a^	3.15±0.18^b^	2.31±0.15^c^	1.22±0.19^c^

^a^ P<0.05 *vs*. Control group

^b^ P>0.05 *vs*. Cisplatin group

^c^ P<0.05 *vs*. Cisplatin group. Values are presented as the mean ± SD.

***Note*:** Con.: control; Cispt: cisplatin; Puer.: Puerarin.

### Effect of puerarin on cisplatin-induced changes of inflammation mediators in the kidney tissues

**Fig 4:**
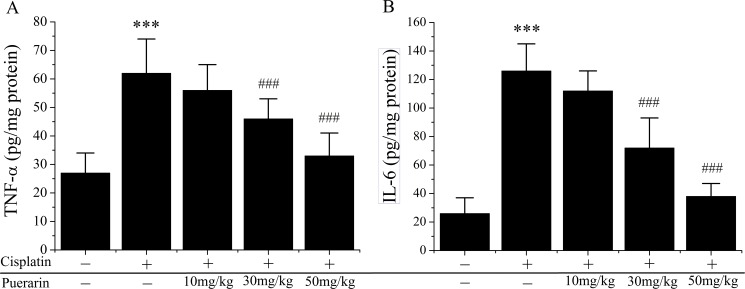
Effects of puerarin on inflammatory cytokines production in kidney tissues of cisplatin treated rats. (A) Tumor necrosis factor-α (TNF-α); (B) Interleukin-6 (IL-6). ***P<0.05 versus control group; ^###^P<0.05 versus cisplatin alone treated group.

Fig 4 showed the changes of TNF-α (Fig 4A) and IL-6 (Fig 4B), two pro-inflammatory cytokines in kidney tissues. Compared with control, cisplatin treatment evoked significant increases in TNF-α (P<0.05) and IL-6 (P<0.05) production, which was reduced by puerarin treatment in a dose-dependent manner (P<0.05). Furthermore, puerarin administration at 50mg/kg reversed cisplatin-upregulated TNF-α and IL-6 levels to normal.

### Effect of puerarin on proteins expression of TLR4 and NF-κB p65 in kidney tissues

**Fig 5:**
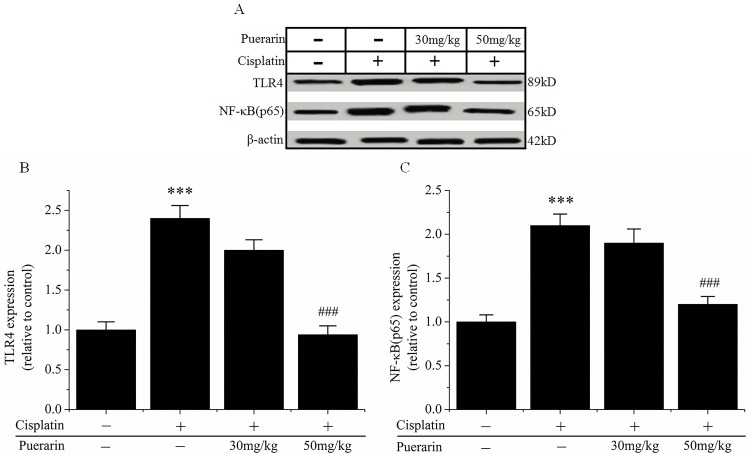
Expression of TLR4 and NF-κB p65 proteins in kidney tissues in each groups. (A) Representative Western blot picture, showing the expression levels of TLR4 and NF-κB p65 proteins. β-actin was used as an internal control. (B) Changes in the expression level of TLR4 protein. (C) Changes in the expression level of NF-κB p65 protein. Data are presented as mean ± SD (n = 4). ***P<0.05 versus control group; ^###^P<0.05 versus cisplatin alone treated group.

Western blot analysis showed that cisplatin administration significantly increased the expression of TLR4 and NF-κB p65 proteins in kidney tissues in cisplatin group. However, in cisplatin + puerarin group cisplatin-induced increases in TLR4 and NF-κB p65 proteins expression were significantly reduced by puerarin treatment (30mg/kg, 50mg/kg) (P<0.05) (Fig 5).

### Effect of puerarin on the action of cisplatin

**Fig 6:**
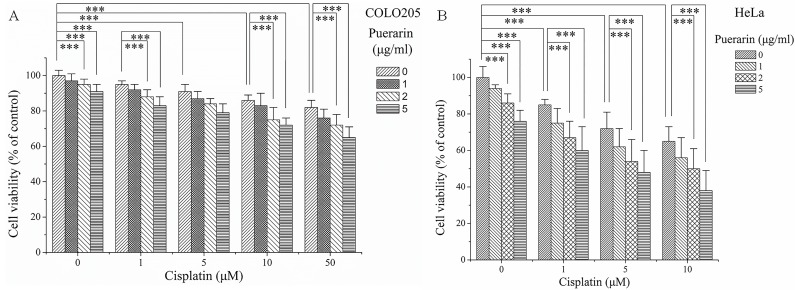
Influence of puerarin on the inhibitory activity of cisplatin in human COLO205 and HeLa cancer cells. ***P<0.05.

Fig 6 demonstrated how puerarin and cisplatin influence tumor cell proliferation. Puerarin alone treatment dose-dependently inhibited the growth of the colon cancer COLO205 cells and cervical cancer HeLa cells (P<0.05). Cisplatin alone treatment also dose-dependently inhibited the growth of the colon cancer COLO205 cells and cervical cancer HeLa cells (P<0.05). When combined with puerarin, the inhibitory effects of cisplatin on tumor cell proliferation were not negatively influenced by puerarin; however, some additive effects might be seen between cisplatin and puerarin.

### Effect of puerarin on NF-κB p65 expression in COLO205 and HeLa cancer cells

**Fig 7:**
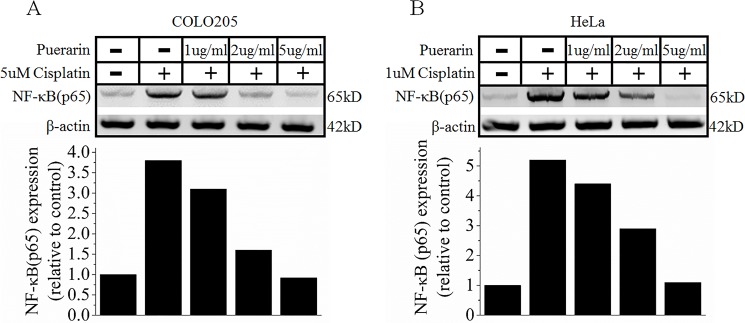
**Western blot analysis about the effects of puerarin on NF-κB signaling pathway in COLO205 colon cancer cells (A) and HeLa cervical cancer cells (B)**.

Western blot analysis showed that compared with control cisplatin treatment induced a significant increase of NF-κB p65 expression in COLO205 and HeLa cells. On the contrary, when puerarin treatment was concomitant with cisplatin treatment, NF-κB p65 expression in COLO205 and HeLa cells presented a significant reduction compared with cisplatin alone treatment (Fig 7A and 7B).

## Discussion

In this study, puerarin was investigated in cisplatin-induced rat nephrotoxic model for the first time. The results showed that cisplatin-induced significant increases in serum urea and creatinine (biomarkers of renal function), lipid peroxidation (MDA level), oxidant (XO), inflammation mediators (TNF-α, IL-6) and significant decreases in some enzymatic and non-enzymatic antioxidants (GSH, GPx, GST, GR, SOD, CAT) in kidney tissues were dose-dependently inhibited by puerarin treatment, which indicated the renal protective effects of puerarin. Findings from histopathological examinations further supported the conclusions from biochemical assays. Cisplatin-induced morphological changes in kidney tissues were ameliorated by puerarin treatment in concentration-dependent manner. At high dose (50mg/kg) puerarin almost completely suppressed cisplatin-induced morphological changes in kidney tissues. Western blot analyses demonstrated that cisplatin-increased TLR4 and NF-κB p65 proteins expression in kidney tissues were reduced by puerarin in concentration-dependent manner, which indicated that it was via inhibiting TLR4/NF-κB pathway puerarin exerted renal protection.

Previous reports investigated the role of puerarin in lead-induced chronic nephrotoxicity [27, 28]. Their results demonstrated that puerarin could attenuate lead-caused renal cells apoptosis and morphologic damages via modulating PI3K/Akt/eNOS pathway [27, 28]. In present study puerarin was studied in cisplatin nephrotoxicity, a frequent devastating side effect of chemotherapy. Puerarin treatment dose-dependently reduced serum urea and creatinine levels increased by cisplatin administration and provided renal protection. The mechanisms of this renal protection were further investigated. The etiology of cisplatin-induced nephrotoxicity was complicated, uncertain and involved multiple factors and numerous signaling pathways. However, the role of oxidative stress, particularly production of reactive oxygen species (ROS) and antioxidant system dysfunction, was well established [29–31]. Cisplatin produced ROS mainly through targeting two subcellular organelles, microsomes [32] and mitochondria [33]. And then, ROS in turn damaged the mitochondria leading to cell death via apoptosis and necrosis in cisplatin-induced nephrotoxicity [30, 34]. ROS might also attack multiple target molecules including lipids, proteins, leading to increased malondialdehyde (MDA) formation and oxidants (XO), decreased.enzymatic and non-enzymatic antioxidants levels or activities (GSH, GPx, GST, GR, SOD, CAT) [10, 35]. In present study, cisplatin-induced increases in formation of MDA and XO oxidase activity in kidney tissues were attenuated by puerarin treatment in concentration-dependent manner (Fig 1). Puerarin treatment also dose-dependently restored renal antioxidants including the levels and /or activities of GSH, GPx, GST, GR, SOD and CAT (Fig 2). These findings strengthened the hypothesis that renal protective effect of puerarin could be attributed to its free radicals scavenging and strong antioxidant properties. However, as puerarin could scavenge ROS and ROS played an important role in the antitumor activity of cisplatin [36], there would be a question that whether puerarin compromise the antitumor activity of cisplatin? Maybe the answer was no. The reasons were as followings. Puerarin could exerted antitumor effect on gastric carcinoma *in vivo* synergistically with 5-fluorouracil, although no combination treatment with cisplatin was *in vivo* investigated at present. ROS did not always play positive role in antitumor action of cisplatin, elevated ROS might also cause cisplatin-resistance and antioxidants might also sensitize tumor cell to cisplatin [37]. For some herbal drugs similar to puerarin harboring renal protective activity against cisplatin nephrotoxicity through antioxidant activity might also increase ROS production in cancer cells to potentiate the antitumor activity of cisplatin, such as curcumin [22, 38–40]. What’s more, results from the tumor cell experiments in present study indicated that puerarin did not compromise the inhibitory effect of cisplatin on tumor cell proliferation *in vitro*. Nevertheless, verification of this postulation and unveiling the real answer warranted further investigations.

A growing body of evidences suggested that inflammation was closely associated with the pathogenesis of cisplatin nephrotoxicity [41]. Over the past decade a plenty of inflammatory mediators, including TNF-α, IL-6, IL-1β, TGF-0058, MIP2, MCP1, were identified to be upregulated in cisplatin-induced nephrotoxicity [41, 42]. However, only TNF-α was verified to play a functional role in cisplatin nephrotoxicity [43]. Ramesh reported that TNF-α was induced by ROS generated by cisplatin and was also an inducer of ROS; Treatment with TNF-α inhibitor alleviated cisplatin-induced renal injury; TNF-α-deficient mice were unsusceptible to cisplatin-induced nephrotoxicity [43]. In present study cisplatin administration induced significant increase of TNF-α and IL-6 levels in kidney tissues, which was greatly decreased by puerarin treatment in dose-dependent manner (Fig 4). These findings indicated that puerarin exerted renal protection through inhibiting inflammation reactions.

Toll-like receptor 4 (TLR4) was a pattern recognition receptor belonging to the TLR family. It recognized the damage-associated molecular pattern molecules (DAMPs) released by damaged tissues to “alert” the immune system to tissue injury [44]. TLR4 expression in murine peritoneal macrophage was increased by cisplatin treatment in vitro [45]. TLR4 was essential to the initiation of intrarenal inflammatory mediators production in cisplatin-induced nephrotoxicity [42]. Remesh also demonstrated that cisplatin synergistically acted with TLR4-specific ligand, lipopolysaccharides (LPS), to produce inflammatory cytokines such as TNF-α and IL-6, thereby leading to nephrotoxicity [46]. In TLR4-deleted mice, cisplatin-induced inflammation and renal injury were significantly reduced compared with wild-type mice [42]. Downstream TLR4 signaling was NF-κB, an critical bridge to inflammatory mediators production [41]. TNF-α production was highly dependent on ROS and NF-κB activation [43]. ROS generated by cisplatin activated the transcript factor NF-κB, which in turn induced the production of proinflammatory cytokines such as TNF-α and IL-6 [47]. Inhibition of NF-κB activation reduced cisplatin nephrotoxicity without affecting its oncolytic action [48, 49], which might be explained by the observation that cisplatin nephrotoxicity was mediated via TNFR2, whereas the anti-tumor effect of TNF-α was mediated by TNFR1. In present study, Western blot results showed that cisplatin treatment increased TLR4 and NF-κB proteins expression in kidney tissues. However, puerarin treatment significantly reduced cisplatin-increased TLR4 and NF-κB proteins expression (Fig 5A–5C); cisplatin-increased TNF-α production in the kidney tissues was also reduced by puerarin treatment. These findings suggested suppression of cisplatin-induced inflammation by puerarin could be partially attributed to its inhibitory effect on TLR4/NF-κB signaling pathway, although other pathways involved in renal inflammation reaction or injury such as MAPKs p38, ERK1/2, and JNK could also be activated by TLR4 and their role in puerarin action couldn’t excluded by presents results [50, 51].

A variety of cells including circulating and resident immune cells and renal parenchymal cells expressed TLR4. Activation of TLR4 in any cells could lead to inflammation reaction and subsequent renal injury [52]. As mentioned above, puerarin treatment could attenuate inflammation through decreasing TLR4 and NF-κB expression, leukocyte infiltration during cisplatin nephrotoxicity. Then, what was the exact site of puerarin action? Maybe the immune cells and parenchymal cells were all targets of puerarin action because previous reports have showed that under other conditions puerarin could inhibit inflammation in immune cells and parenchymal cells in some other organs [53, 54]. However, in order to clearly elucidate the targets and mechanisms of puerarin action future further investigations were warranted.

According to the results mentioned above, puerarin might be a potential adjuvant agent for inhibiting cisplatin nephrotoxicity. However, as treating cancer was the main purpose of cisplatin, it would be unpractical for future puerarin application in cancer patients if puerarin interfere in the action of cisplatin. So, in order to find out whether puerarin interfered the action of cisplatin, human colon cancer COLO205 cells and cervical cancer HeLa cells were employed and in vitro cell culture experiments were performed. Results indicated that puerarin alone treatment exerted inhibitory effects on tumor cell proliferation *in vitro*, consistent with previous reports [13, 15–17]. What’ more, when combined with cisplatin, the *in vitro* inhibitory effects of cisplatin on tumor cell proliferation were not negatively influenced by puerarin. These findings suggested that puerarin might harbor no interference in cisplatin action *in vitro*. Protein analyses by Western blot also provided further supportive evidence for this conclusion (Fig 7). NF-κB, a ubiquitous transcription factor, was constitutively activated in many human cancers [55, 56]. NF-κB activation was associated with tumor promotion, progression and chemoradiotherapy resistance, and so it was proposed as a target for cancer therapy [57]. Targeting NF-κB activation was a strategy to overcome resistance to chemotherapy [58]. In present study, cisplatin treatment increased NF-κB p65 protein expression in both COLO205 and HeLa cells (Fig 7), which might resulted in potential cisplatin resistance [58, 59]. However, puerarin treatment greatly reduced cisplatin- increased NF-κB p65 protein expression in COLO205 and HeLa cells (Fig 7), which might exert inhibitory effects on NF-κB signaling pathway and facilitate cisplatin treating cancer more efficiently according to current point of view [58].

## Conclusion

In summary, findings in this investigation suggested that puerarin harbored renal protection by inhibiting oxidative stress and inflammation in cisplatin-induced nephrotoxicity. Inhibition of TLR4/NF-κB signaling pathway was involved in the renal protective activity of puerarin. Puerarin showed no inhibitory influence on the antitumor action of cisplatin while protecting kidney from cisplatin toxicity. On the contrary, puerarin further promoted the antitumor action of cisplatin via inhibiting cisplatin-induced NF-κB activation.

## Supporting information

S1 File “data set.rar”: Figures of the experiment results. There were seven figures in the “data set.rar” file. Caption for each figure was presented below the paragraph in which the figure was cited.
(RAR)
